# Facing the Danger Zone: The Use of Ultrasound to Distinguish Cellulitis from Abscess in Facial Infections

**DOI:** 10.1155/2014/935283

**Published:** 2014-01-16

**Authors:** Dywanda L. Lewis, Christine J. Butts, Lisa Moreno-Walton

**Affiliations:** Section of Emergency Medicine, Department of Medicine, Louisiana State University Health Sciences Center, New Orleans, LA 70112, USA

## Abstract

Physical exam alone is often insufficient to determine whether or not cellulitis is accompanied by an abscess. Bedside ultrasound can be a valuable tool in ruling out suspected abscess by allowing direct visualization of a fluid collection. The proximity of the infection to adjacent structures can also be determined, thus aiding clinical decision making. Patients with cellulitis near the eye and nose are of particular concern due to the adjacent facial structures and the anatomy of the venous drainage. Accurately determining the presence or absence of an associated abscess in these patients is a crucial step in treatment planning. The purpose of this report is to (1) emphasize the benefits of bedside ultrasound when used in conjunction with the physical exam to rule out abscess; (2) demonstrate the utility of bedside ultrasound in planning a treatment strategy for soft tissue infection; (3) depict an instance where ultrasound detected an abscess when computed tomography (CT) scan did not.

## 1. Background

The physical exam findings of cellulitis include skin erythema, edema, and warmth. An abscess is suspected on physical exam when a tender, fluctuant area is palpated within this area of cellulitis [1]. If an abscess is diagnosed, incision and drainage must be performed. Cellulitis, however, can be medically managed with antibiotics. Distinguishing the two conditions is of utmost importance in order to properly treat the patient and to spare patients with cellulitis an invasive, uncomfortable procedure.

### 1.1. Soft Tissue Ultrasound Basics

Ultrasound uses sound waves generated by the ultrasound probe. The sound waves strike objects in the body and bounce back stronger or weaker depending on the composition of the tissue the wave strikes. The ultrasound machine then displays the image as black (hypoechoic) to represent fluid, white (hyperechoic) to represent dense hard structures, and shades of gray to represent tissue compositions between these two extremes.

### 1.2. Soft Tissue Examination by Ultrasound

**Figure 1:**
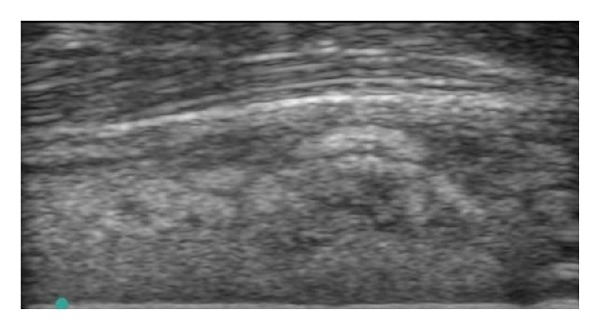
Cellulitis: subcutaneous tissue swelling, increased fluid accumulation, and fat lobules form a cobblestoned appearance [2].

**Figure 2:**
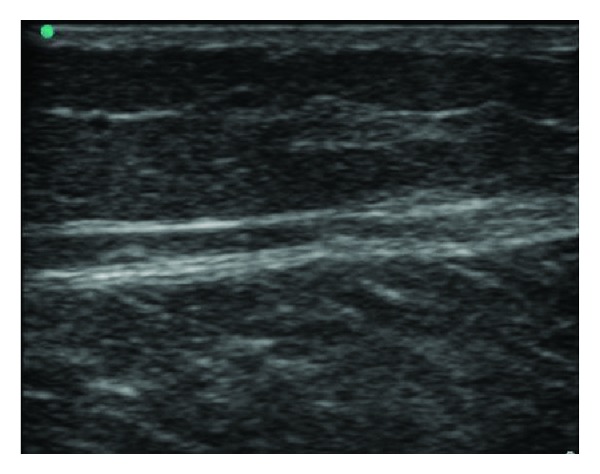
Normal soft tissue: well-organized tissue layers—skin subcutaneous layer, and connective tissue layers.

**Figure 3:**
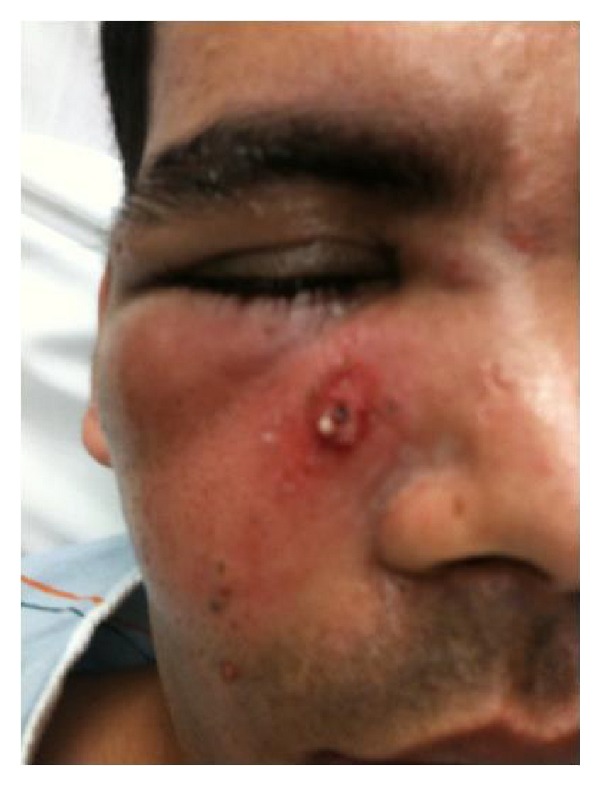
Photograph of case patient taken on day of presentation.

When there is diagnostic uncertainty regarding the presence of an abscess, imaging is indicated to assist in the diagnosis. Imaging options may include CT scan (usually with IV contrast) or ultrasound. CT scan is considered by many to be the diagnostic “gold standard” for diagnosing abscesses. However, CT scans are not always available, expose patient to ionizing radiation and IV contrast, and are expensive [3]. Ultrasound, although user dependent, is usually readily available in the emergency department (ED) and can be rapidly performed, providing real-time assessment and identification of surrounding structures [4]. Figures 1, 2, and 3 demonstrate classic ultrasound images of normal tissue, cellulitis, and abscess [1, 2]. Once a fluid collection is identified, ultrasound can characterize the size and depth of an abscess and can be used to directly guide incision and drainage [4, 5]. Furthermore, ultrasound can be used at bedside and presents no risk to the patient.

## 2. Case Presentation

**Figure 4:**
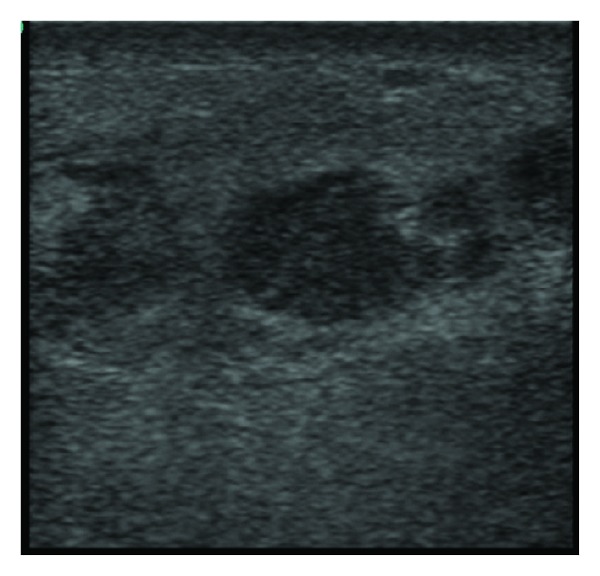
Abscess: hypoechoic area surrounded by soft tissue swelling.

**Figure 5:**
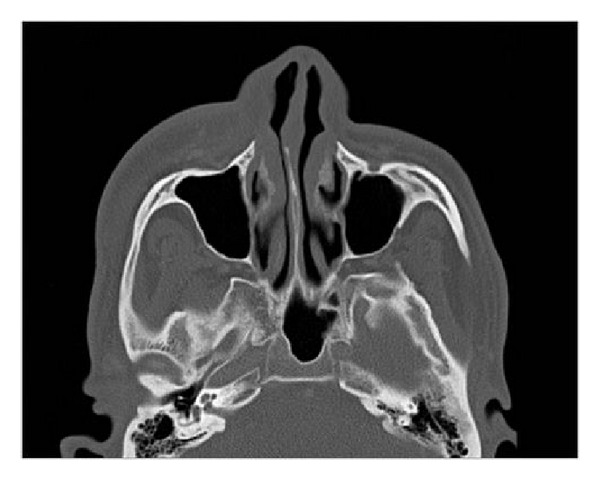
CT scan of case patient. There is soft tissue swelling overlying right maxilla. No definitive abscess is identified.

**Figure 6:**
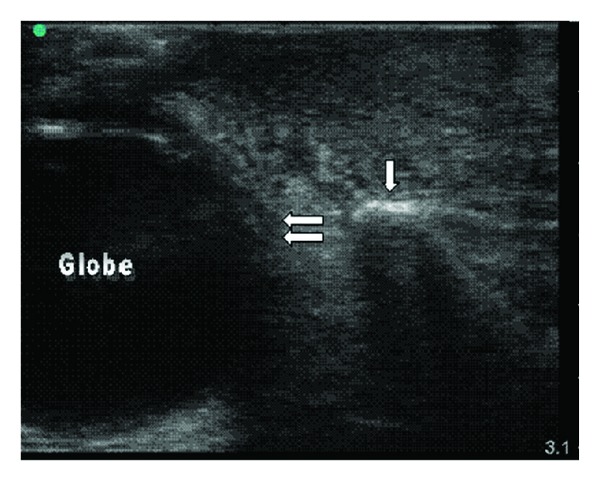
Bedside ultrasound of the case patient using linear probe, transverse view. The hyperechoic area labeled with a single arrow represents the maxilla. There is an area of cellulitis within the subcutaneous tissue above the maxilla which is distinguished by the hypoechoic streaks of fluid interrupting the normal tissue organization. The edge of the globe of the eye is labeled with double arrows.

**Figure 7:**
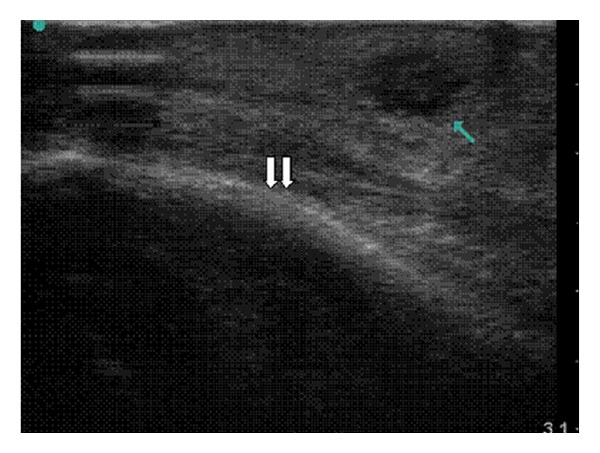
Bedside ultrasound of case patient using linear probe, sagittal view. A distinct abscess (single arrow) is apparent in the subcutaneous tissue overlying the maxilla (double arrows). The single arrow points to the abscess, which is distinguished as a well-circumscribed area of hypoechoic fluid.

A 31-year-old male presented to the ED with right facial swelling that began 2 days ago. He reported chronic swelling in this area for several months. Pain and swelling became worse two days ago when he squeezed and manipulated the area to try to get it to drain. His pertinent past medical history included facial cellulitis with abscess in the same area 1.5 years ago requiring incision and drainage by a specialist. He denied fever, chills, headache, neck stiffness, vision changes, or pain with eye motion. He denied prior surgeries, allergies, and medications. Vital signs were within normal range with blood pressure of 122/71, heart rate of 84, respiratory rate of 18, and temperature of 98.4 degrees Fahrenheit. The physical exam was pertinent to right periorbital and malar erythema, edema with overlying pustule with scant serous exudate, and tenderness to palpation (Figure 4). Pertinent labs include white blood cells 9.3, hemoglobin 15.2, hematocrit 44.8, platelets 179, sodium 141, K 4, chloride 105, bicarbonate 28, glucose 112, blood urea nitrogen 9, creatinine 0.79, erythromycin sedimentation rate 5, and C-reactive protein 2.07. Maxillofacial CT with contrast showed preseptal cellulitis with no specific abscess (Figure 5). Bedside ultrasound revealed a small, soft tissue abscess in close proximity to the globe (Figures 6 and 7). A facial specialist was consulted to perform the incision and drainage procedure. The patient was hospitalized and treated with IV antibiotics for 2 days. The infection improved and the patient was subsequently discharged without further complication.

## 3. Discussion 

Soft tissue infection is a common presenting complaint in the ED. The severity of soft tissue infections among patients is highly variable and may range from localized, superficial, and minute to extensive, intrusive to nearby structures, and progressive to systemic involvement. Clinicians frequently must assess whether or not an abscess is present within the infected tissue. The appropriate management plan depends on this assessment. Diagnostic accuracy becomes even more important where facial infections in or near the “danger zone,” the triangle formed by the bridge of the nose and the corners of the mouth, are involved [6, 7]. Venous drainage in this area forms a communication with the brain via the superior and inferior ophthalmic veins, which empty into the cavernous sinus, creating the potential for facial infections to spread to the brain and cause serious complications such as vision loss, ophthalmoplegia, meningitis, encephalitis, intracranial abscess, sepsis, seizure, coma, and death [6, 7].

When the diagnosis of abscess is suspected but is not clinically evident, computerized tomography with intravenous contrast is the imaging modality most frequently utilized [3]. The use of bedside ultrasound to evaluate soft tissue infection improves the diagnostic accuracy of the physical exam for the presence or absence of an abscess [3, 8]. Furthermore, ultrasound can be used when CT scan is not available. It is noninvasive, spares exposure to radiation, and can be used to guide the incision and drainage of an abscess [3, 5].

## 4. Conclusion

When assessing patients with soft tissue infection, physical exam alone can be unreliable in detecting an occult abscess [8]. Imaging methods such as CT are frequently relied upon to distinguish an underlying fluid collection. In the case presented in this report, CT scan failed to reveal the abscess. Ultrasound is an advantageous imaging modality, not only because it poses no risk to the patient, but also because it rapidly expands the information gleaned from the physical exam. As this case emphasizes, ultrasound is a convenient, noninvasive, accurate tool that allows for rapid decision making and effective treatment of soft tissue infection.
